# Tautological formal explanations: does prior knowledge affect their satisfiability?

**DOI:** 10.3389/fpsyg.2023.1258985

**Published:** 2023-09-28

**Authors:** Ivan Aslanov, Ernesto Guerra

**Affiliations:** Center for Advanced Research in Education, Institute of Education, Universidad de Chile, Santiago, Chile

**Keywords:** explanations, formal explanations, prior knowledge, category labels, tautology

## Abstract

It is known that formal explanations with categorical labels are more satisfying than explicit tautologies. However, would they still be more satisfying if they are implicitly tautological themselves? In two experiments, we compared the degree of satisfaction between tautological formal explanations, explicit tautologies, and proper explanations. Additionally, we examined whether participants knew the correct definitions for the labels used in the formal explanations. Finally, we asked whether cultural and linguistic differences can play a role in the treatment of formal explanations with categorical labels. To this end, the first experiment involved Chilean students (*N* = 50), and the second experiment involved Russian students (*N* = 51). It was found that formal explanations, despite their intentional tautology, were still rated as more convincing compared to explicit tautologies (but less convincing than proper explanations). Furthermore, this effect did not depend on participants’ previous knowledge (the label’s definitions) or linguistic and cultural background. Taking all this into account, we consider this effect as a relatively universal psychological phenomenon and relate our findings to existing theories of formal explanations.

## 1. Introduction

### 1.1. Background

Categories encompass explanatory structures that enable the understanding of the reasons an observed phenomenon possesses specific features (Rehder and Hastie, 2001; Keil, 2006; Carey, 2009; Lombrozo, 2009; Walker et al., 2014; Prasada, 2017). Different types of explanations may appeal to distinct information, such as functional or mechanistic aspects, which can influence the level of satisfaction (Shaw et al., 2003; Lombrozo, 2010; Liquin and Lombrozo, 2022; Sulik et al., 2023). However, formal explanations hold a unique position among the various types of explanations. They explain the presence of specific properties in an object by referring to its essential nature (Prasada, 2017). For example, “Why does this creature fly?” – “Because it is a bird.” Some researchers argue that such explanations may appear to be mere tautologies (Gelman et al., 2018) and lack any new information (Hemmatian and Sloman, 2018). Despite seemingly relying only on a categorical label (e.g., “bird”), these explanations are often considered more satisfying than explicit tautologies (e.g., Giffin et al., 2017). Prasada and colleagues have demonstrated that formal explanations are more persuasive when applied to principled properties of a category (Prasada and Dillingham, 2006; Prasada et al., 2008; Prasada and Dillingham, 2009; Haward et al., 2018, 2021). According to this theory, formal explanations are persuasive when they establish principled connections between a category and an exemplar, such as having four legs being a principled property for a dog, whereas wearing a collar is not (Prasada, 2017). Thus, some researchers argue that formal explanations, though seemingly devoid of meaning on their own, indicate communal knowledge of the category (Sloman and Rabb, 2016; Hemmatian and Sloman, 2018; Hemmatian et al., 2019; Sloman, 2022) or suggest an underlying cause yet to be discovered (Gelman et al., 2018). Others consider formal explanations as a distinct mode of explanation that emphasizes principled attributes (Rivera et al., 2023) and corresponds to human essentialism, a significant aspect of cognition (Ahn et al., 2001; Rangel and Keller, 2011; Cimpian and Salomon, 2014). While researchers investigate the potential tautological nature of such explanations and seek plausible reasons for their paradoxical persuasiveness, it is important to note two features of previous experiments in the literature.

First, despite the debates surrounding informativeness and tautology, the effects of formal explanations are not always tested on complete tautologies. From a common interpretation, to which we shall also align in this article, tautology is an “unnecessary repetition, usually in close proximity, of the same word, phrase, idea, argument” (Oxford English Dictionary, 2023). Thus, examples like “that flies because it is a bird” (Gelman et al., 2018), “he holds a poorly-paid job because he is an immigrant” (Vasilyeva and Lombrozo, 2020), or “Fido has four legs because he is a dog” (Rivera et al., 2023) are technically not tautologies. The word “bird” does not equate to “being able to fly,” the word “immigrant” does not equate to “having a low-paying job,” and the word “dog” does not equate to “having four legs.” Such explanations, could be regarded as uninformative, and it is unsurprising that the authors perceive their resemblance to tautologies (Gelman et al., 2018). However, since they are still not tautological in the strict sense of the word, it is entirely anticipated that participants in the experiments perceive such explanations as more satisfying when compared to tautological explanations (e.g., “Because it is s a bird” vs. “Because it flies”; Gelman et al., 2018). Hemmatian and Sloman (2018) use examples of formal explanations that are close to tautologies, but they do not compare them to explicit tautologies, and their examples involve fictional categories. While the persuasiveness of formal explanations over explicit tautologies has been demonstrated before, it is necessary to investigate whether this effect extends to formal explanations involving real categories that are themselves (implicitly) tautological. An example of such an explanation would be: “Alcohol promotes cancer because it is a carcinogen” (the word “carcinogen” literally means something that promotes cancer). This aspect is crucial because people are highly sensitive to tautological explanations and circular argumentation, perceiving them as unconvincing (Baum et al., 2008; Corriveau and Kurkul, 2014; Mercier et al., 2014; Mills et al., 2019).

Second, many of these experiments employ either simplistic categories (“dogs,” “cars,” etc.) or artificial categories with provided definitions. Consequently, participants are always aware of the category to which the label refers in a formal explanation. This allows them to supplement the explanation of a specific example with categorical information based on their prior knowledge during interpretation (Aslanov et al., 2022). However, a direct comparison has not been made to determine whether the satisfiability of a formal explanation depends on participants knowing the definition of the label (e.g., if they are unaware of the definition of “carcinogen”). While participants who are familiar with a definition possess the necessary information to detect a “hidden” tautology, those unfamiliar with the definition may be persuaded by the scientific terminology used in the explanations, which itself reinforces their persuasiveness (Weisberg et al., 2008; Pennycook et al., 2015; Weisberg et al., 2015).

Finally, the existing body of research has not yet directly assessed the potential cultural and linguistic effects on the evaluation of formal explanations. Previous experiments have mainly focused on native English speakers (e.g., Gelman et al., 2018; Hemmatian and Sloman, 2018; Rivera et al., 2023) leaving a gap in our understanding of how this effect manifests in different languages and populations. In this context, the present research aims to contribute to enhancing our understanding of the possibilities for generalizing the effect by comparing results cross-culturally and cross-linguistically. By doing so, we can begin to understand whether cultural and linguistic factors may influence the evaluation of formal explanations.

### 1.2. The present study

In the present study, we hypothesized, that tautological formal explanations (e.g., “Alcohol promotes cancer because it is a carcinogen”) will be perceived as more satisfactory compared to explicitly tautological explanations (e.g., “Alcohol promotes cancer because it is a substance that promotes cancer”), but as less satisfactory compared to “real” explanations (e.g., “Alcohol promotes cancer because its metabolic products damage cell DNA”) (*Hypothesis 1*). We also assumed that the satisfactoriness of tautological formal explanations will vary based on participants’ prior knowledge of the concept under consideration. Specifically, tautological formal explanations will be rated as more satisfactory when participants have no or incomplete prior knowledge of the concept compared to when they possess accurate prior knowledge about the concept (*Hypothesis 2*). Additionally, if the observed effects are inherent cognitive phenomena, they should be observable across different languages and cultures (*Hypothesis 3*). To investigate this, the study was conducted among Spanish speakers in Chile and Russian speakers in Russia.

By testing these hypotheses, we aimed at enhancing our understanding of the evaluative and persuasive aspects of tautological formal explanations and examine the role of prior knowledge in their perceived satisfactoriness. Furthermore, the cross-linguistic and cross-cultural exploration would contribute to determining the generalizability of these effects.

## 2. Experiment 1

### 2.1. Participants

50 Chilean students were recruited through advertisements on Instagram and the local network of Universidad de Chile (M = 22.6, SD = 3.2 years; 27 females, 20 males, 1 demigender, 1 non-binary person and 1 person who chose not to specify their gender). All of them read and signed an informed consent. They received an amount equivalent to approximately 5 USD (in Chilean pesos) for their participation.

### 2.2. Materials and design

We designed 24 items across 4 domains: Biology, Chemistry, Social Sciences, and Linguistics (6 items in each domain). For each item, three variants of explanations were constructed (see Supplementary Appendix): explicitly tautological (or “control condition”), tautological formal (“label condition”), and proper explanations (“explanation condition”). Each tautological formal explanation was constructed according to the following scheme: a specific representative of a kind (e.g., alcohol / neon / cactus) possesses a distinct property (causes cancer / does not undergo chemical reactions / has fleshy tissues for water retention) *because* it belongs to a kind of phenomena unified by this property (is a carcinogen / is an inert gas / is a succulent). In a few instances where naming a specific object posed difficulty (such as when describing an atom that is an ion), we contextually clarified that we were referring to some individual entity (“*This* atom possesses an electric charge because it is an ion”). Two fillers were also added to each domain (1 filler for “label” and 1 for “explanation” conditions), which were obviously incorrect statements (e.g., “Chinese is the most widely spoken modern language, with over 1.3 billion speakers, because it has hieroglyphic writing”). We did this in order to reduce the possibility of participants judging the satisfiability of an explanation solely based on its formal characteristics, as well as to balance the average number of characters in the statements across the three conditions. Thus, in total, this section consisted of 32 questions (24 items and 8 fillers).

In addition, we developed a multiple-choice questionnaire in which participants were required to select the correct definition for each item from three possible options. For instance, to test one’s understanding of the term “polyglot,” we provided the following question: “Which of these definitions best fits the word ‘polyglot’?” and supplied three answers for selection: a) A person who speaks many languages, b) A person trained as a linguist, and c) A person who has spoken two languages since childhood. This section consisted of 24 questions (in accordance with the number of items from the previous section). We used Latin square experimental design with one within-subject and within-items factor (type of explanation) with three levels, and one within-subject between-item factor with two levels (known vs. unknown). The levels of this second factor emerge from the multiple-choice questionnaire.

### 2.3. Procedure

Each participant was assigned to one (out of three) experimental lists, presenting all items in one condition each, and the same number of each condition. This allowed us to have all items presented in every condition across participants. Participants were instructed to evaluate the explanations provided by different people for various phenomena. In this stage, participants were asked, “How satisfactory do you find the following explanation?” We used the term satisfaction, because it has been previously used to study label effects (see Gelman et al., 2018). The question was accompanied by the explanation that they were required to evaluate. Ratings were given on a seven-point scale, ranging from 1 to 7 (from “completely unsatisfactory” to “completely satisfactory”). The presentation of items was fully randomized. After participants evaluated the explanations, they proceeded to the second stage, during which they had to choose the correct definition. The experiment was conducted online using the Open Lab platform and was built using the lab.js editor.

### 2.4. Data analysis

Before analysis, we checked the quality of the data by plotting the frequency of the responses (from 1 to 7) at the participant level. This is to ensure that we detect whether any participant responded mindlessly by pressing the same digit all the time. While, in principle participants should respond 8 trials as 1 (for the control condition), and respond 8 trials as 7 (for the explanation condition), it is less clear whether their responses for the label condition will tend to either of the extremes of the scale. However, our hypothesis dictates that this condition will fall between in between the extremes values. Thus, we tolerated up to 50% of responses equal to 7 (14 or more responses equal to 7). This criterion resulted in the exclusion of 2 participants in the Chilean sample. Accuracy was not considered an exclusion criteria since we were interested in having a range of known and unknown concepts. Yet, no participant in the Chilean sample exhibited accuracy at or below chance (accuracy min-max values = 54–100%).

As per preregistration,^1^ after collecting 50 participants and before conducting any inferential analysis in our data, we check whether we would have enough statistical power (> = 0.8) to detect the effects necessary to address our hypotheses. This was achieved via simulations of 1,000 experiments with parameters (mean and SD for each condition) extracted from our actual data. Since two participants were already excluded from the initial sample, we conducted two simulations, one with 48 participants and 24 items, as well as another with 100 participants and 24 items. These simulations show that with 48 participants we had enough power to test our first hypothesis, but not the second hypothesis (power for the difference between the label condition and the explanation condition was 1; power for the difference between the label condition and the control condition was 0.92; and power for the difference between the label condition when knowing the concept compared to when not knowing the concept was 0.1). However, these simulations also showed that even with 100 participants we would not have enough power to test our second hypothesis (power for the difference between the label condition when knowing the concept compared to when not knowing the concept = 0.24). Since our stop rule was set to 100 participants in the preregistration, and even with that sample size we would not have enough power to the test our second hypothesis, we settle for 48 participants.

Inferential analysis was carried out using a mixed-effects regression approach with the lme4 (Bates et al., 2015) and lmerTest (Kuznetsova et al., 2017) packages in R. Hierarchical regressions allow the accommodation of the random variability at the participant and item levels without the need of aggregating data. Consequently, the inferential analysis was based on a linear mixed-effect regression with type of explanation, knowledge and their interaction as fixed effects. Since the label condition was the condition of most interest, we set this level of our first factor as reference group (or intercept) using a treatment contrast. For the knowledge factor, we used a sum contrast such as that we can evaluate the difference between label-when-known and label-when-unknown via the knowledge predictor in the regression analysis. Finally, the random structure of the models included random intercepts for participants and items, as well as random slope for the knowledge factor, the type of explanation factor, and their interaction at the participant level, and but only the type of explanation as random slope at the item level, since knowledge is a between-item factor. All our data, scripts and experimental materials are available at https://osf.io/zjsf2/.

### 2.5. Results

**Figure 1:**
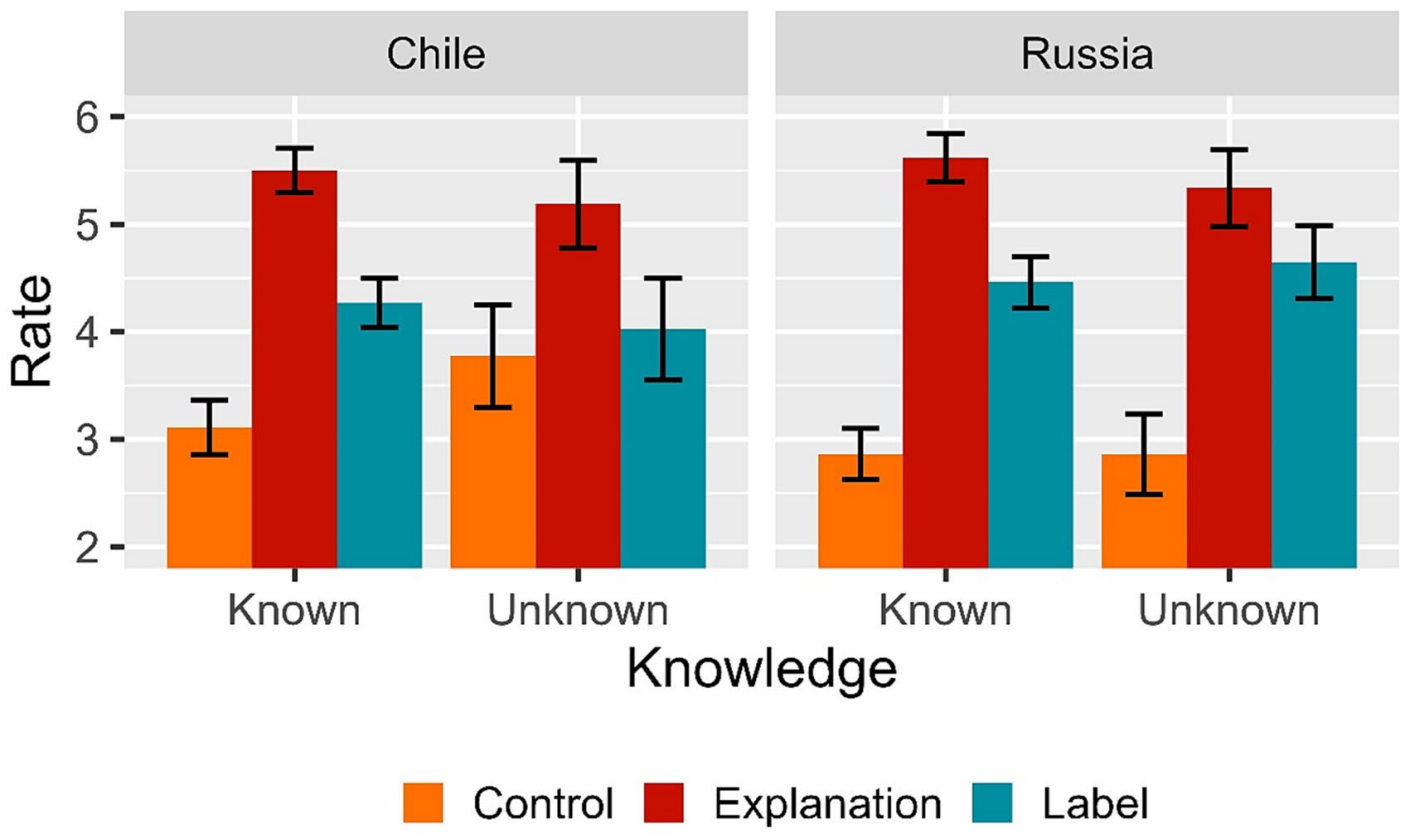
Mean rate as a function of experimental condition (Control, Explanation and Label) and Knowledge (Known vs. Unknown) for both Experiment 1 (*Left panel*) and Experiment 2 (*Right panel*). Error bars represent 95% confidence intervals adjusted for within-subject designs.

Table 1 presents the results of the linear mixed-effect regression analysis for Experiment 1. As it can be seen, we observed an overall difference between the ratings for the label condition and those for the explanation conditions (Estimate = 1.403, *se* = 0.216, *t*-value = 6.50, *p* <. 001) and between the ratings for the label condition and those for the control conditions (Estimate = −0.883, *se* = 0.220, *t*-value = −4.01, *p* < 0.001). Figure 1 (*left panel*) shows the pattern of the results in Experiment 1. As predicted, we found the lower rates for the control condition, the highest rates in the explanation condition and the label condition fell in between, being statistically different than the other conditions as shown by the results of the linear mixed effect regression.

**Table 1:** Results of the linear mixed-effect regression analysis for Experiment 1.

	Estimate	*se*	*t*-values	*p*-values	
(Intercept)	4.111	0.223	18.46	<0.001	***
Knowledge Effect on Label	0.251	0.133	1.89	0.063	.
Label vs. Explanation	1.403	0.216	6.50	<0.001	***
Label vs. Control	−0.883	0.220	−4.01	<0.001	***
Knowledge Effect on Label vs. Explanation	−0.180	0.173	−1.04	0.299	
Knowledge Effect on Label vs. Control	−0.221	0.169	−1.31	0.193	

Signif. codes: “***” <0.001; “.” <0.1.

## 3. Experiment 2

### 3.1. Participants

A sample of 51 Russian students were recruited through advertisements among several university teachers and students (M = 20.6, SD = 3.0 years; 38 females, 13 males). Students received credit for courses or payment for participation. All of them read and sign an informed consent.

### 3.2. Materials, design, procedure, and data analysis

The materials, design, procedures, and data analysis were identical to those used in Experiment 1, except that all materials and instructions were in the Russian language. Consequently, we also check data quality using the same criteria as in the Chilean sample, which resulted in the exclusion of 3 participants. We therefore simulated 1,000 experiments with 48 participants, 24 items and the means and SD observed in the Russian sample, as well as the same simulations but with 100 participants. As for the Chilean sample, we found enough power for our first hypothesis even with 48 participant (power for the difference between the label condition and the explanation condition was 1; power for the difference between the label condition and the control condition was 1; and power for the difference between the label condition when knowing the concept compared to when not knowing the concept was 0.1), but, even with simulations for 100 participant, we did not found enough statistical power for our second hypothesis (with 100 participants: power for the difference between the label condition when knowing the concept compared to when not knowing the concept = 0.14). Thus, we also applied our stop rule here, as we did for Experiment 1.

### 3.3. Results

Table 2 presents the results of the linear mixed-effect regression analysis for Experiment 2. As in Experiment 1, we found an overall difference between the ratings for the label condition and those for the explanation conditions (Estimate = 0.976, *se* = 0.197, *t*-value = 4.94, *p* <. 001) and between the ratings for the label condition and those for the control conditions (Estimate = −1.868, *se* = 0.208, *t*-value = −8.96, *p* < 0.001). Figure 1 (*right panel*) shows the pattern of the results in Experiment 2. As predicted, we found the lower rates for the control condition and the highest rates in the explanation condition, while for the label condition rating appeared in between, being statistically different than the other two conditions, result confirmed through the linear mixed effect regression.

**Table 2:** Results of the linear mixed-effect regression analysis for Experiment 2.

	Estimate	*se*	*t*-values	*p*-values	
(Intercept)	4.542	0.195	23.31	<0.001	***
Knowledge effect on label	−0.138	0.103	−1.33	0.186	
Label vs. Explanation	0.976	0.197	4.94	<0.001	***
Label vs. Control	−1.868	0.208	−8.96	<0.001	***
Knowledge effect on label vs. Explanation	0.169	0.137	1.23	0.221	
Knowledge effect on label vs. Control	0.285	0.144	1.97	0.050	.

Signif. codes: “***” <0.001; “.” <0.1.

## 4. Exploratory analysis

**Figure 2:**
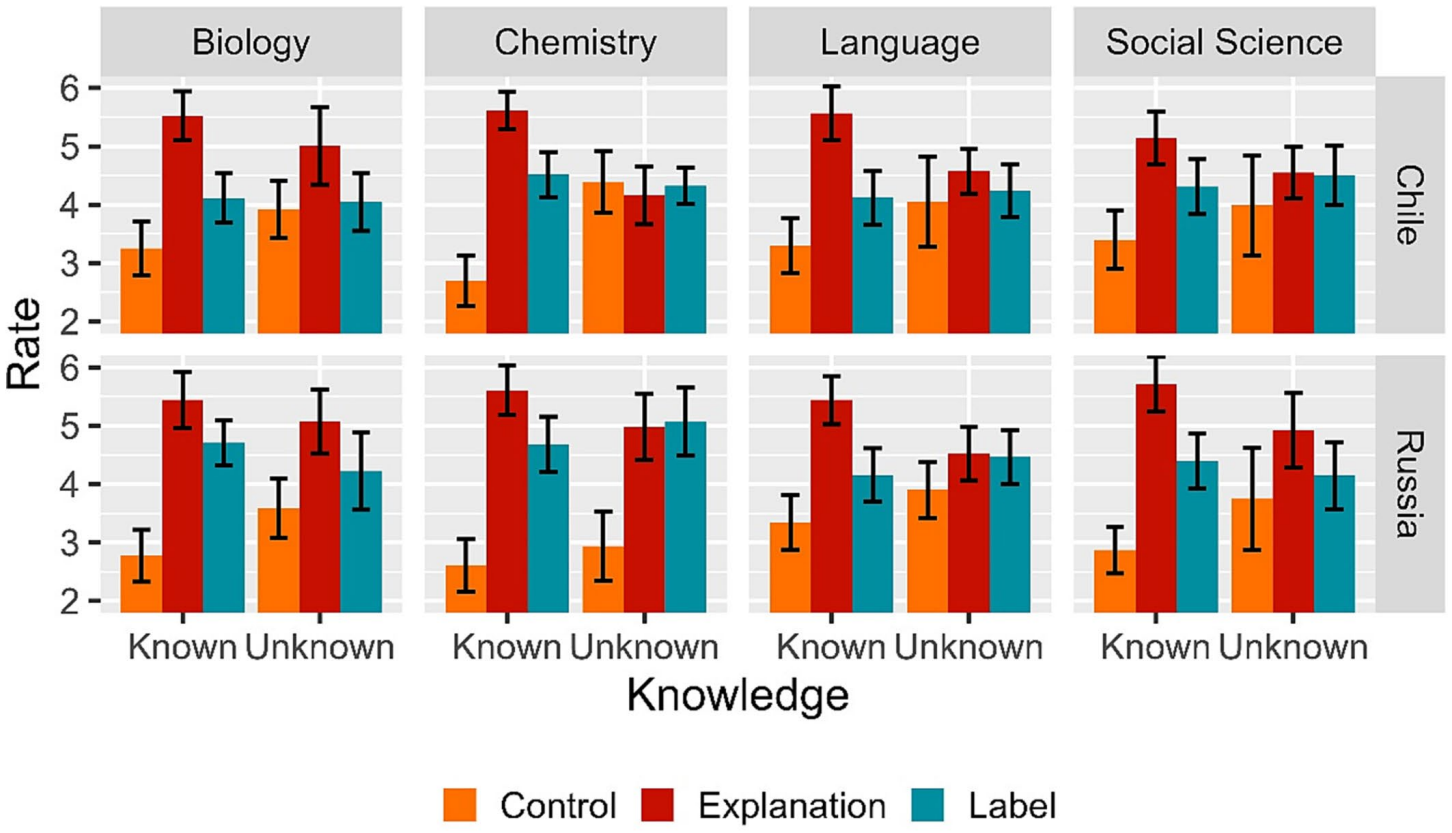
Mean rate as a function of experimental condition (Control, Explanation and Label), Knowledge (Known vs. Unknown), and Discipline (Biology, Chemistry, Language, and Social Sciences) for both Experiment 1 (*Left panel*) and Experiment 2 (*Right panel*). Error bars represent 95% confidence intervals adjusted for within-subject designs.

As stated in our preregistration, although we did not have a specific hypothesis about any potential differences between the four disciplines (i.e., Chemistry, Biology, Social Science, and Language), we include this variable in an exploratory analysis. This analysis was based on descriptive statistics initially (see Figure 2), followed by inferential analysis with “Discipline” as a control variable in the regression models. Based on Figure 2, we chose Biology as reference group, since it is the group that shows the pattern that is most similar to the overall pattern of results. The inclusion or exclusion of this control variable was determined via model comparison using the *anova* R function. The results indicate that in neither of the experiments did a model with disciplines as a predictor perform better than a model without them (Chilean sample: *χ*^2^ = 13.957, *df* = 18, *p* = 0.73; Russian sample: *χ*^2^ = 21.361, *df* = 18, *p* = 0.26), suggesting that disciplines had no effect in the way participants rated the explanations.

## 5. General discussion

The current study investigated, in two distinct populations, whether the effect previously observed in formal explanations extends to cases involving full tautologies, and explored whether this effect depends on prior knowledge about the explained concepts, and on cultural and linguistic background. Our first hypothesis predicted that participants would perceive tautological formal explanations as more satisfying compared to explicit tautologies, but less satisfying compared to proper explanations. This hypothesis was confirmed in both the Russian and Chilean samples. The second hypothesis predicted that prior knowledge would influence this effect; we supposed that participants who could correctly indicate the definition of a label would also evaluate tautological formal explanations as less satisfying compared to those who indicated incorrect definitions. Our results do not support that hypothesis. Using the observed means and SDs, we calculated the power for that effect simulating 100 participants on each sample. The results of those simulations show that even with that large sample size we would not be able to detect the effect more than 10% of the time, which can be interpreted as the effect being too small to be relevant, or if the effect is large enough to be relevant, it cannot be detected by our task. While we acknowledge that the replication of this study on a larger sample could theoretically yield different outcomes, nevertheless, the results of these simulations indicate that even with a twofold increase in the sample size, the detection of the effect remains improbable. Therefore, we favor the first interpretation and believe that both Russian and Chilean participants evaluated tautological formal explanations as more satisfying than explicitly tautological explanations regardless of whether they were familiar with the label definition or not. These findings are somewhat counterintuitive in nature.

One might assume that a person who knows the definition of the word “carcinogen” would have a greater chance of understanding that “Alcohol promotes the formation of cancerous tumors in the body because it is a *carcinogen”* is a tautology and would rate its satisfiability lower. An alternative viewpoint could predict that, on the contrary, if a person is unfamiliar with the definition, they cannot grasp the meaning of the formal explanation and therefore perceive it as unsatisfactory. However, none of this occurred. According to our data, if an explanation relies on an appeal to category membership, it will be perceived as more satisfying than an explicit tautology – even if the individual possesses the necessary knowledge to notice the tautological nature or even if the person is unaware of or misunderstands the definition of the categorical label. Taking this into account, along with the fact that these findings hold true for two different populations (Russian and Chilean), and that the disciplinary domains (Biology, Chemistry, Social science, and Language) did not contribute to the effect, we interpret our results as showing a universal psychological phenomenon, which might be linked to the formal-logical structure of the explanation itself rather than its content.

According to Prasada’s theory (Rivera et al., 2023), the satisfiability of formal explanations is based on establishing principled connections between a category and an exemplar of that category: a formal explanation will be deemed satisfactory if it explains features that are “principled” to the category. Therefore, this assumes the presence of adequate categorical knowledge. However, our results show that individuals who were unable to select the correct definition out of three options still rated the tautological formal explanation as more satisfying than explicitly tautological explanation. Thus, knowledge of a category and corresponding principled features did not become a factor that influenced the satisfiability of the explanations.

Alternatively, it is possible that our method has limitations in its capacity to assess knowledge about the label. For instance, if a participant believed that a “polyglot” is “a person trained as a linguist,” the characteristic of “speaking many languages” may still be considered principled by such a respondent. Since we used multiple-choice questions for knowledge assessment, we cannot rule out this possibility, nor confirm it. Future research could employ an alternative approach by testing the knowledge of definitions using open-ended questions or controlling distractors in used multiple-choice questions to ensure they do not contain the same principled features as the correct response. This would allow for a more comprehensive examination of participants’ understanding and provide insights into the robustness and generalizability of the observed effects. Given these limitations, we must still note that our data does not support Prasada’s theory.

Other theories suggest that formal explanations can indicate potential knowledge that can be used to explain a feature. This knowledge can be held either among members of a social community (Hemmatian and Sloman, 2018) or within the inherent nature of the object itself (Gelman et al., 2018). Therefore, despite the participant lacking the necessary information, and the explanation itself not providing it, tautological formal explanations are evaluated as more satisfying than explicitly tautological explanations because the former provides directions for further exploration or enhances the credibility of the facts that are subject to explanation. We suppose that our data can be considered as consistent with these theories. Furthermore, the average rating of the tautological formal explanation in both populations is close to 4 out of 7 points, which falls around the middle of the 7-point scale. This could be interpreted as follows: although the explanations are not unsatisfactory, they still lack sufficient information to be considered fully comprehensive.

Thus, tautological formal explanations are not explanations in the full sense of the word (when compared to real explanations). Yet, participants in both experiments do not consider as unsatisfactory as explicit tautologies, regardless of their knowledge of label meaning. Therefore, either tautological formal explanations are not tautologies from a cognitive perspective (they mean “more” to individuals than just formal logic), or participants apply a heuristic when assessing the satisfactoriness of such explanations. The latter would imply that in certain cases, formal explanations indeed possess the ability to reduce the sense of uncertainty by adding new information. Let us examine a formal (yet non-tautological) explanation: “Why does that [pointing at a bird] fly?” – “That flies because it is a bird” (Gelman et al., 2018). Despite the fact that the inquirer evidently perceives a bird before them (rendering this explanation minimally informative), there remains the possibility for it to be (potentially) some other flying creature, thereby causing this explanation to slightly reduce the level of uncertainty. A tautological formal explanation cannot fulfill this function: “The famous archeologist Heinrich Schliemann could speak at least 15 languages, because he was a polyglot.” Schliemann could not be anyone else if he spoke multiple languages, yet the heuristic enhances the satisfactoriness of such an explanation based on its formal structure. Whatever the case is, future research should address these hypotheses empirically, for instance, by including an experimental manipulation with labels that are able to reduce uncertainty (“That animal feeds milk to their offspring because it is a goat”), against others that do not (“That animal feeds milk to their offspring because it is a mammal”). Alternatively, the persuasiveness of a formal (non-tautological) explanation may stem from the sense engendered by the logicality of a valid syllogism. For instance, “birds can fly, this entity is a bird, therefore this entity can fly.” However, even in this instance, a tautological formal explanation likely emerges as more satisfactory due to the heuristic nature, as they lack false propositions and, by formal attributes, resemble statements that are inclined to encompass valid syllogisms. Nevertheless, regardless of the specifics, we suppose that for some reason, non-tautological formal explanations exhibit a certain degree of persuasiveness to individuals, prompting individuals to extend their trust in formal explanations even to instances involving tautological formal explanations (which are not syllogisms and are incapable of reducing uncertainty). It would appear that this tendency extends even to those instances where individuals are unfamiliar with the definition of the respective label.

Despite the fact that the effect has been demonstrated on two culturally and linguistically different samples and across four different domains, some limitations need to be acknowledged. Our study was focused on students, and it is important to ascertain whether the effect can manifest in other social groups. Additionally, considering that explanation evaluation is influenced by both individual differences (Sulik et al., 2023) and learning motivation (Liquin and Lombrozo, 2022), it is necessary to investigate their interaction with the described effect. Taking this into consideration, with the acknowledgment that there is still room for future research, we conclude that the satisfiability of formal explanations is not dependent on prior knowledge of label definitions and manifests itself across different linguistic populations and thematic domains.

## Data availability statement

The datasets presented in this study can be found in online repositories. The names of the repository/repositories and accession number(s) can be found at: https://osf.io/zjsf2/.

## Ethics statement

The studies involving humans were approved by Comité de Ética de la Investigación en Ciencias Sociales y Humanidades. The studies were conducted in accordance with the local legislation and institutional requirements. The participants provided their written informed consent to participate in this study.

## Author contributions

IA: Conceptualization, Data curation, Investigation, Methodology, Validation, Writing – original draft, Writing – review & editing. EG: Formal analysis, Methodology, Software, Validation, Visualization, Writing – original draft, Writing – review & editing.

## Footnotes

^1^ https://osf.io/3st4k
