# Supplementary material for: Tautological formal explanations: does prior knowledge affect their satisfiability?
Source: Front Psychol. 2023 Sep 28;14:1258985. doi: 10.3389/fpsyg.2023.1258985 (PMC10568452; doi:10.3389/fpsyg.2023.1258985)
Supplement: Supplementary file 1 [file Table_1.docx]

Supplementary Material

# Appendix. Examples of experimental materials

| **Condition** | **Domain** | | | |
| --- | --- | --- | --- | --- |
|  | **Biology** | **Chemistry** | **Social science** | **Linguistics** |
| **Label** | Alcohol promotes the formation of cancerous tumours in the body because it is a *carcinogen*. | This atom possesses an electric charge because it is an *ion*. | Richard Dawkins does not believe in the existence of God because he is an *atheist* | The famous archaeologist Heinrich Schliemann could speak at least 15 languages, because he was a *polyglot*. |
| **Explanation** | Alcohol promotes cancer because the products of its metabolism damage the genetic material of cells. | This atom possesses an electric charge because the number of electrons in its composition exceeds the number of protons. | Richard Dawkins does not believe in the existence of God because there is no scientific evidence to support it. | The famous archaeologist Heinrich Schliemann could speak at least 15 languages because he invented a special way to learn languages quickly. |
| **Control** | Alcohol promotes cancer because it is the substance that causes cancerous tumors. | This atom possesses an electric charge because it is an electrically charged atom. | Richard Dawkins does not believe in the existence of God because he does not believe in the existence of God. | The famous archaeologist Heinrich Schliemann could speak at least 15 languages, because he spoke many languages. |
